# Association between chronic hepatitis B infection and COVID-19 outcomes: A Korean nationwide cohort study

**DOI:** 10.1371/journal.pone.0258229

**Published:** 2021-10-05

**Authors:** Seong Hee Kang, Dong-Hyuk Cho, Jimi Choi, Soon Koo Baik, Jun Gyo Gwon, Moon Young Kim

**Affiliations:** 1 Department of Internal Medicine, Yonsei University Wonju College of Medicine, Wonju, Republic of Korea; 2 Regenerative Medicine Research Center, Yonsei University Wonju College of Medicine, Wonju, Republic of Korea; 3 Division of Cardiology, Department of Internal Medicine, Yonsei University Wonju College of Medicine, Wonju, Gangwon, Republic of Korea; 4 Division of Endocrinology and Metabolism, Department of Internal Medicine, Korea University College of Medicine, Seoul, Republic of Korea; 5 Department of Transplantation and Vascular Surgery, Korea University College of Medicine, Seoul, Republic of Korea; Seoul National University College of Medicine, REPUBLIC OF KOREA

## Abstract

**Background/Aims:**

We measured the association between underlying chronic hepatitis B (CHB) and antiviral use with infection rates among patients who underwent severe acute respiratory syndrome coronavirus 2 (SARS-CoV-2) testing.

**Methods:**

In total, 204,418 patients who were tested for SARS-CoV-2 between January and June 2020 were included. For each case patient (n = 7,723) with a positive SARS-CoV-2 test, random controls (n = 46,231) were selected from the target population who had been exposed to someone with coronavirus disease 2019 (COVID-19) but had a negative SARS-CoV-2 test result. We merged claim-based data from the Korean National Health Insurance Service database collected. Primary endpoints were SARS-CoV-2 infection and severe clinical outcomes of COVID-19.

**Results:**

The proportion of underlying CHB was lower in COVID-19 positive patients (n = 267, 3.5%) than in COVID-19 negative controls (n = 2482, 5.4%). Underlying CHB was associated with a lower SARS-CoV-2 positivity rate, after adjusting for comorbidities (adjusted odds ratio [aOR] 0.65; 95% confidence interval [CI], 0.57–0.74). Among patients with confirmed COVID-19, underlying CHB tended to confer a 66% greater risk of severe clinical outcomes of COVID-19, although this value was statistically insignificant. Antiviral treatment including tenofovir and entecavir was associated with a reduced SARS-CoV-2 positivity rate (aOR 0.49; 95% CI, 0.37–0.66), while treatment was not associated with severe clinical outcomes of COVID-19.

**Conclusions:**

Underlying CHB and antiviral agents including tenofovir decreased susceptibility to SARS-CoV-2 infection. HBV coinfection did not increase the risk of disease severity or lead to a worse prognosis in COVID-19.

## Introduction

Coronavirus disease 2019 (COVID-19) is an emerging respiratory disease caused by severe acute respiratory syndrome coronavirus 2 (SARS-CoV-2) emerged in Wuhan, China, and rapidly spread to other regions [1]. The COVID-19 pandemic has claimed 196,107,976 laboratory-confirmed cases and 4,195,176 deaths globally as of July 28, 2021. The World Health Organization reported an overall case-fatality rate of 2.14% [2].

Previous studies have revealed COVID-19 likely induces liver injury, and 14–53% of COVID-19 patients developed hepatic dysfunction, particularly individuals with severe disease [3]. Recent reports have shown that about 2–11% of patients with COVID-19 had underlying chronic liver disease [4]. HBV viruses which cause a global infection and threat public health. The global prevalence of HBsAg is about 3.9% [5]. As SARS-CoV-2 and HBV both can cause liver damage [6], enhancing our understanding of the risk of SARS-CoV-2 infection in patients with chronic hepatitis B (CHB) is urgently required.

To date, most studies that have assessed the impact of HBV on SARS-CoV-2 have been performed on patients from China, due to the high prevalence of HBV within the country. In a large series of patients from Wuhan, China, it was observed that 2.1% of patients possessed underlying CHB, although this was defined solely by the presence of HBsAg, and no data on antiviral therapy were provided. However, a recent letter showed that the HBV rates of those with COVID-19 remained between 0–1.3%, which is a lower incidence than that of corresponding individuals of similar ages in the general population. The authors reported that their data indicated the presence of an inverse relationship between HBV and COVID-19 [7]. Based on this result, one can speculate about the reason a low incidence of HBV infection in patients hospitalized with COVID-19. However, to date, no studies have reported whether HBV antiviral therapy affects COVID-19 incidence and outcomes.

Given these reports, we hypothesized that underlying CHB and current use of antiviral agent might influence susceptibility to SARS-CoV-2 infection and COVID-19 outcomes. Using a large-scale, population-based, nationwide cohort in Korea, we measured potential associations between the current use of CHB antiviral therapy with SARS-CoV-2 infection rates in patients who underwent SARS-CoV-2 testing.

## Material and methods

### Data sources

The Korean Government has committed to providing mandatory and complementary health insurance for all patients with COVID-19 during the pandemic. This COVID-19 cohort study is a retrospective, nationwide case-control study using Korean National Health Insurance Service COVID database (NHIS-COVID DB) that includes all Koreans who were tested for SARS-CoV-2 through services facilitated by Korean National Health Insurance Service (NHIS), Korea Centers for Disease Control and Prevention, and the Health Insurance, Review & Assessment Service of Korea between January 1, 2020 and June 4, 2020 as a result of medical or Korea Centers for Disease Control referral. From this data, we obtained the demographic information, previous health screening results, three years of complete healthcare records, test results for SARS-CoV-2, and clinical outcomes of COVID-19 from January 1, 2015 to August 18, 2020 [8]. The detailed study protocol used was previously described [9, 10]. All patient related records used in our study were anonymized to ensure confidentiality.

### Study population

**Fig 1:**
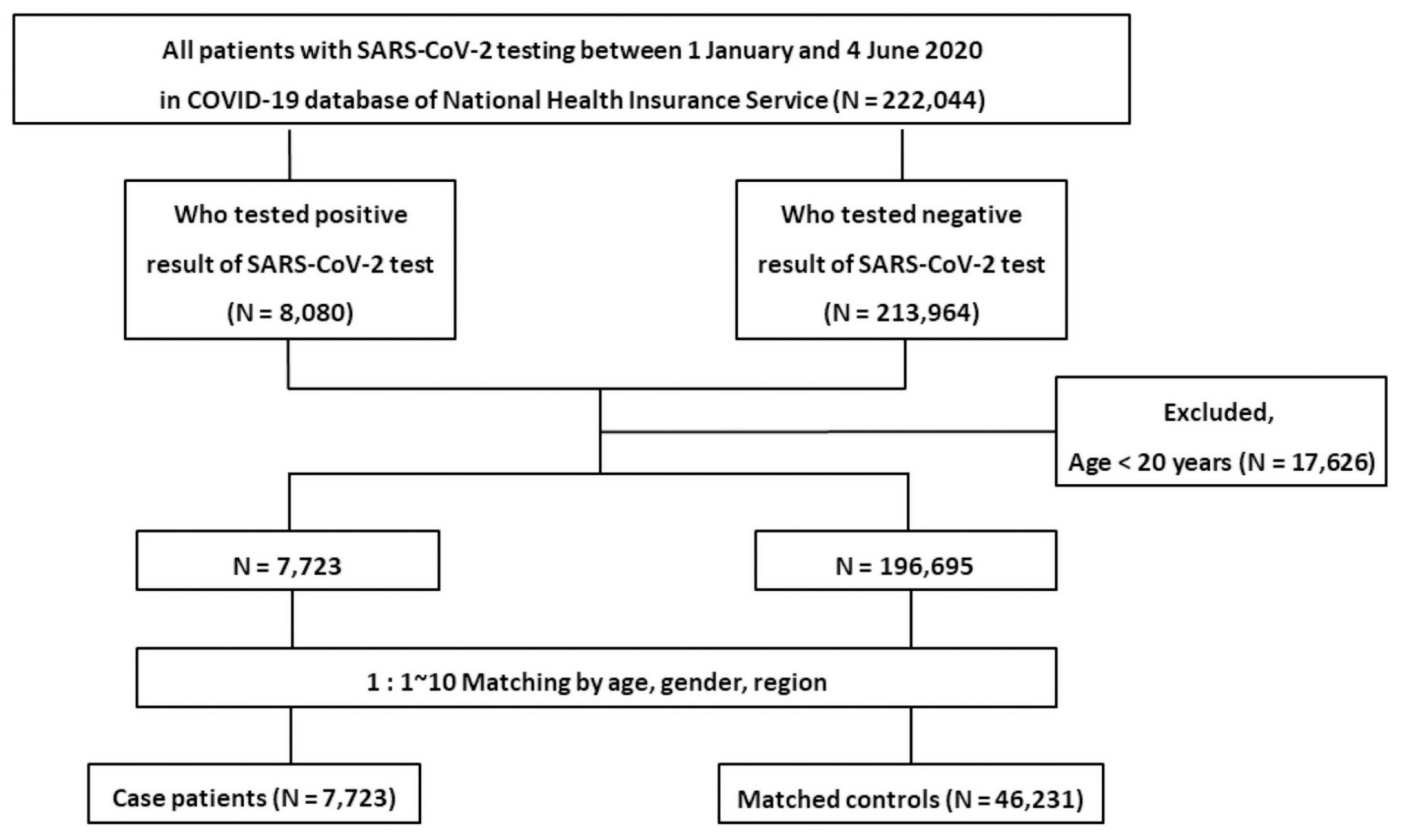
A flow chart of the inclusion and exclusion of patients from the Korean nationwide cohort.

Among the 222,044 patients tested for SARS-CoV-2 test between January 1, 2020, and June 4, 2020, patients younger than 20 years of age were excluded (n = 17,626). Therefore, 204,418 patients were enrolled in the study. A flow diagram describing patient inclusion and exclusion is shown in Fig 1.

The date of the first SARS-CoV-2 test of each patient was defined as the entry date (individual index date) of the study. SARS-CoV-2 infection was confirmed by a positive real-time reverse transcriptase PCR analysis of pharyngeal and nasal swab cultures, according to World Health Organization recommendations [11]. Case patients were defined as individuals with positive SARS-CoV-2 test results. For each case patient, random controls were selected from the target population who had been exposed to someone with COVID-19 but had a negative SARS-CoV-2 test result. We merged claim-based data from the NHIS collected January 1, 2015 and August 18, 2020, and extracted information regarding the sex, age and region of residence of included individuals at the date of test. A history of underlying disease (diabetes mellitus, hypertension, dyslipidemia, cardiovascular disease, chronic obstructive pulmonary diseases (COPD), chronic kidney disease, chronic hepatitis B/C and liver cirrhosis) was confirmed by the assignment of at least two claims within a year using appropriate International Classification of Diseases, 10th revision (ICD-10) codes from insurance eligibility data. Patients with the following comorbidities were excluded: chronic hepatitis C, alcohol related liver disease/liver cirrhosis, coinfection with human immunodeficiency virus (HIV) and hepatocellular carcinoma (HCC). The institutional review board of Korea University Anam Hospital approved this study (2020AN0292), and the requirement for informed consent was waived.

Chronic liver disease was diagnosed based on ICD-10 codes (Chronic hepatitis B: B18.0, B18.10; Chronic hepatitis C: B18.2; Liver cirrhosis: K74.0, K74.1, K74.2, K74.6; Alcohol hepatitis and alcohol related liver cirrhosis: K70.0, K70.1, K70.2, K70.3, K70.4; HCC: C22.0). We identified patients taking chronic hepatitis B antiviral agents (adefovir, clevudine, telbivudine, entecavir, besifovir and tenofovir) who were prescribed the drugs more than twice within the 30-day period that preceded the index date [12].

### Study outcomes

The primary outcome assessed was a positive laboratory test result for SARS-CoV-2. Secondary outcomes considered were the presence of severe COVID-19 (intensive care unit admission, administration of invasive ventilation, mortality) and mortality.

### Statistical analysis

We performed exact matching in age, sex and region of residence between case and control groups and randomly selected up to 10 control subjects per 1 case patient. Variables were demonstrated as frequencies (percentages). Differences in demographic, clinical, and laboratory variables between case and control groups were compared using a conditional logistic regression model for matched data. Multiple conditional logistic regression analysis for matched data was performed to determine the association between the presence of CHB, or antiviral treatment for CHB, and SARS-CoV-2 infection after adjustment for hypertension, diabetes mellitus, dyslipidemia, liver cirrhosis and economic income. To evaluate the association between the antiviral treatment for CHB and clinical outcomes in case patients, we used multiple logistic regression model adjusting age, sex, hypertension, diabetes mellitus, dyslipidemia, liver cirrhosis and economic income. SAS Enterprise Guide software version 7.1 (SAS Institute, Cary, NC, USA) was used to perform the statistical analysis. A two-sided p-value < 0.05 was considered statistically significant.

## Results

### Baseline characteristics

The number of patients with a positive SARS-CoV-2 test result was 7,723, and the number of matched controls was 46,231. Baseline characteristics of the entire study subjects are displayed in Table 1. There was no difference in age distribution, sex, and region of residence when cases and controls were assessed due to exact matching. However, case patients were had lower rates of comorbidities and other concurrent medications than control individuals.

**Table 1:** Demographic and clinical characteristics of patients with COVID-19 (case patients) and subjects with negative test result (matched controls).

	Case Patients		Matched Controls		P value*
	(n = 7,723)		(n = 46,231)		
	N	(%)	N	(%)	
Age group					-
*20–29*	2,059	(26.7)	10,572	(22.9)	
*30–39*	834	(10.8)	7,264	(15.7)	
*40–49*	1,037	(13.4)	7,417	(16.0)	
*50–59*	1,569	(20.3)	8,253	(17.9)	
*60–69*	1,200	(15.5)	6,282	(13.6)	
*70–79*	618	(8.0)	3,466	(7.5)	
*80-*	406	(5.3)	2,977	(6.4)	
Male	3,056	(39.6)	21,366	(46.2)	-
Region					-
*Seoul*	515	(6.7)	5,150	(11.1)	
*Daegu*	5,036	(65.2)	20,108	(43.5)	
*Gyeonggi*	435	(5.6)	4,350	(9.4)	
*Gyeongbuk*	933	(12.1)	8,583	(18.6)	
*Others*	804	(10.4)	8,040	(17.4)	
Medical history					
*Hypertension*	1,914	(24.8)	13,991	(30.3)	< .001
*DM*	845	(10.9)	6,152	(13.3)	< .001
*Dyslipidemia*	1,834	(23.7)	12,571	(27.2)	< .001
*CVD*	1,468	(19.0)	10,822	(23.4)	< .001
*COPD*	86	(1.1)	1,438	(3.1)	< .001
*CKD*	62	(0.8)	1,505	(3.3)	< .001
Liver disease					
*Liver cirrhosis*	70	(0.9)	1,168	(2.5)	< .001
*Chronic hepatitis B*	267	(3.5)	2,482	(5.4)	< .001
*Chronic hepatitis C*	77	(1.0)	540	(1.2)	0.301
Medication†					
*Statin*	1,162	(15.0)	8,503	(18.4)	< .001
*Antiplatelet agents*	682	(8.8)	5,562	(12.0)	< .001
*Anticoagulants*	74	(1.0)	1,135	(2.5)	< .001

*p-value by conditional logistic regression model.

^†^prescribed more than twice within 30days before the index date.

Abbreviations: CKD, chronic kidney disease; COPD, chronic obstructive pulmonary disease; CVD, cerebrovascular disease; DM, diabetes mellitus.

### Association of SARS-CoV-2 test result positivity with CHB

The proportion of patients with underlying CHB were lower in case patients (n = 267, 3.5%) than control individuals (n = 2482, 5.4%; P < 0.001). Table 2 shows the unadjusted and adjusted odds ratio (OR) from the logistic regression analysis. Underlying CHB was significantly associated with a reduced SARS-CoV-2 test positivity rate, the association was consistently significant after adjusting for comorbidities including hypertension, diabetes mellitus, dyslipidemia, liver cirrhosis and economic income (adjusted OR [aOR] 0.65; 95% CI, 0.57 to 0.74; P < 0.001).

**Table 2:** Association between history of CHB and risk of COVID-19.

	Total	N	(%)	Unadjusted OR	95% CI	P value	Adjusted OR	95% CI	P value
**History of CHB**									
For COVID-19*				0.58	0.51–0.67	< .001	0.65	0.57–0.74	< .001
*Matched controls*	46,231	2,482	(5.4)						
*Cases*	7,723	267	(3.5)						
For death**				1.51	0.84–2.74	0.172	1.29	0.63–2.63	0.483
*Survival*	7,486	255	(3.4)						
*Death*	237	12	(5.1)						
For severity**				1.66	1.10–2.52	0.016	1.34	0.84–2.14	0.218
*Mild*	7,243	241	(3.3)						
*Severe*	480	26	(5.4)						

*By multiple conditional logistic regression model including hypertesion, DM, dyslipidemia, and liver cirrhosis as independent variables.

**By multiple logistic regression model including age, gender, hypertesion, DM, dyslipidemia and liver cirrhosis as independent variables.

Abbreviations: CHB, chronic hepatitis B; CI, confidence interval; OR, odds ratio.

Regarding CHB medication, patients of the population took various antiviral agents including adefovir, entecavir, telbivudine, and tenofovir. The proportion of patients with CHB who took antiviral drugs was 0.6% in case patients and 1.3% in controls (Table 3). Antiviral agent medication for CHB was significantly associated with a reduced SARS-CoV-2 test positivity rate after adjusting for comorbidities (aOR, 0.49; 95% CI, 0.37 to 0.66; P < 0.001). In a subanalysis that assessed individual antiviral agents, tenofovir (aOR, 0.50; 95% CI, 0.34 to 0.74; P < 0.001) and entecavir (aOR, 0.44; 95% CI, 0.27 to 0.71; P = 0.001) were associated with the reduced SARS-CoV-2 test positivity rates, but adefovir was not.

**Table 3:** Association between use of CHB medication and risk of COVID-19.

	Case Patients (n = 7,723)		Matched Controls (n = 46,231)		Odds ratio for COVID-19					
	N	(%)	N	(%)	Unadjusted OR	95% CI	P value	Adjusted OR	95% CI	P value
**CHB medication**	50	(0.6)	585	(1.3)	0.52	0.38–0.69	< .001	0.49	0.37–0.66	< .001
Adefovir	4	(0.1)	17	(0.04)	1.66	0.55–5.06	0.370	1.78	0.56–5.68	0.328
Entecavir	18	(0.2)	252	(0.5)	0.45	0.28–0.74	0.001	0.44	0.27–0.71	0.001
Telbivudine	1	(0.01)	5	(0.01)	-	-	-	-	-	-
Tenofovir	29	(0.4)	361	(0.8)	0.52	0.35–0.76	0.001	0.50	0.34–0.74	<0.001

* p-value by conditional logistic regression model.

Abbreviations: CHB, chronic hepatitis B; CI, confidence interval; OR, odds ratio.

### Association between clinical outcomes of SARS-CoV-2 infection and CHB

Among 7,723 case patients, 480 (6.2%) patients with severe COVID-19 were identified and 237 (3.1%) patients died during hospitalization. Regarding underlying CHB, 26 (5.4%) patients with severe COVID-19 and 12 (5.1%) fatalities occurred in patients with CHB. Table 2 includes data that demonstrates the association between CHB and both SARS-CoV-2 infection and COVID-19 clinical outcome. The presence of underlying CHB was associated with increased risk of severe COVID-19 in univariate analysis (OR, 1.66; 95% CI, 1.10 to 2.52; P = 0.016). However, neither association was significant after adjusting for the presence of comorbidities (aOR, 1.34; 95% CI, 0.84 to 2.14; P = 0.218). In addition, underlying CHB was not associated with enhanced COVID-19 mortality risk (aOR, 1.29; 95% CI, 0.63 to 2.63; P = 0.483). Also, since CHB itself is an important cause of cirrhosis in the Korean population, it may affect the clinical outcome. Therefore, a subanalysis was performed without adjustment for cirrhosis, except for patients with other causes of cirrhosis. As a result, the effect of CHB on severity (aOR, 1.25; 95% CI, 0.80 to 1.96; P = 0.336) and death (aOR, 1.16; 95% CI, 0.59 to 2.29; P = 0.662) was maintained, as shown in S1 Table in S1 File.

Results of our analysis of COVID-19 outcomes in patients currently using antiviral agents for CHB are included in Table 4. Among 50 patients that were prescribed antiviral agents, severe COVID-19 occurred in 4 (8.0%) patients with 2 (4.0%) fatalities. Risk of mortality (aOR 1.52; 95% CI, 0.29 to 8.07; P = 0.625) and severe COVID-19 (aOR 1.09; 95% CI, 0.36 to 3.25; P = 0.880) in patients who took antiviral agents and those who had never been prescribed antivirals did not differ.

**Table 4:** Odds ratios for outcomes associated with use of CHB medication in COVID-19 patients.

	**Severe (n = 480)**		**Mild (n = 7,243)**		**Odds ratio for severe outcome**					
	N	(%)	N	(%)	Unadjusted OR	95% CI	P value	Adjusted OR	95% CI	P value
**CHB medication**	4	(0.8)	46	(0.6)	1.32	0.47–3.67	0.600	1.09	0.36–3.25	0.880
Adefovir	0	(0.0)	4	(0.1)	-	-	-	-	-	-
Entecavir	1	(0.2)	17	(0.2)	-	-	-	-	-	-
Tenofovir	3	(0.6)	26	(0.4)	1.75	0.53–5.79	0.362	1.81	0.49–6.75	0.377
	**Death (n = 237)**		**Survival (n = 7,486)**		**Odds ratio for mortality**					
	N	(%)	N	(%)	Unadjusted OR	95% CI	P value	Adjusted OR	95% CI	P value
**CHB medication**	2	(0.8)	48	(0.6)	1.32	0.32–5.46	0.702	1.52	0.29–8.07	0.625
Adefovir	0	(0.0)	4	(0.1)	-	-	-	-	-	-
Entecavir	0	(0.0)	18	(0.2)	-	-	-	-	-	-
Tenofovir	2	(0.8)	27	(0.4)	2.36	0.56–9.95	0.244	4.83	0.75–31.17	0.098

* p-value by multiple logistic regression model.

Abbreviations: CHB, chronic hepatitis B; CI, confidence interval; OR, odds ratio.

### Association between clinical outcomes of SARS-CoV-2 infection and CHB-associated cirrhosis

We also analyzed whether CHB-associated cirrhosis was associated with poor outcomes in COVID-19. Among 37 patients with CHB-associated cirrhosis, 7 (26.9%) had a severe COVID-19 and 3 (25.0%) died. Although CHB-related cirrhosis only accounted for a small proportion of patients, the presence of cirrhosis was not associated with the severity (aOR, 1.47; 95% CI, 0.50 to 4.38; P = 0.487) nor death (aOR, 1.01; 95% CI, 0.19 to 5.39; P = 0.999) as outcomes of COVID-19 (S2 Table in S1 File).

## Discussion

In this Korean nationwide cohort, we investigated whether underlying CHB and antiviral agent treatment for CHB increased susceptibility to SARS-CoV-2 infection in 53,954 patients who underwent SARS-CoV-2 testing. We also assessed whether outcomes of COVID-19 were affected by CHB or CHB treatment. We found that underlying CHB decreased susceptibility to SARS-CoV-2 infection; however, CHB was not associated with severe outcomes of COVID-19. Furthermore, antiviral agents including tenofovir also decreased susceptibility to SARS-CoV-2 infection.

The identities of host factors that affect risk of infection and clinical COVID-19 infection remain unknown. One brief report described COVID-19 in a patient with preexisting immune dysfunction from coinfection with both HIV and HCV. The report indicated that the patient had a persistently negative SARS-CoV-2 RNA test but a delayed plasma antibody response [13]. CHB may be an important cause of viral hepatitis and pre-existing liver disease comorbidity that affects COVID-19 outcomes. Regarding the underlying CHB, a recent brief report in China showed a surprisingly low prevalence of chronic HBV in COVID-19 cases admitted to the hospital; the HBV rate in the general population aged 47–51 was 7%–11%, while the HBV rate of those infected with COVID-19 ranged from 0% to 1.3% [7]. In agreement with this finding, our study revealed that underlying CHB was significantly associated with a low SARS-CoV-2 test positivity rate. These results could be related to the presence of either host or external factors.

With regard to host factors, chronic HBV infection causes various immune-modulatory effects that include weak or absent virus-specific T-cell reactivity [14]. This phenomenon is described as an “exhaustion” state and is characterized by poor effector cytotoxic activity, impaired cytokine production, and sustained expression of multiple inhibitory receptors. It may affect the cellular ability to respond to other viruses, thus leading to lower susceptibility to SARS-CoV-2 infection. Despite these assumptions, it is unclear whether this is a simple epidemiological “misconnection” or real a consequence of immune dysregulation.

External factors including antiviral treatments, such as tenofovir, decreased susceptibility to SARS-CoV-2 infection, but did not affect the clinical prognosis in this study. The clinical evidence for the influence of SARS-CoV-2 and HBV coinfection on the severity of COVID-19 is limited. In a study by He *et al*. [15] out of 571 COVID-19 patients, enrolled were 15 (2.6%) patients with HBV infection, of which only 3 (20%) received anti-HBV therapy (entecavir). They were observed to have a lower risk of severe events (0% vs 6.47%; P = 0.30). However, the effect of antiviral treatment on prognosis was not analyzed. Additionally, in a cohort of 326 confirmed COVID-19 patients, of which 20 (6.1%) had HBV coinfection, Chen *et al*. [16] reported that there were no differences in discharge rate and length of stay. A few studies have reported conflicting results. A study by Wu *et al*. [17] involving 70 cases of coinfection, indicated that the proportion of severely ill patients was higher than that in a non-HBV infection group (32.86% vs 15.27%). Although several studies to date were limited with a small sample size of cases with pre-existing HBV infection, these studies appeared to suggest that in most cases, HBV coinfection did not increase the risk of disease severity nor lead to worse prognosis in COVID-19. However, in several published studies, the number of patients with HBV coinfection was small, especially because fewer patients were taking antiviral drugs, which may have influenced the results. A recent large-scale cohort study conducted in Spain found that the incidence of SARS-CoV-2 infection in CHB treated with tenofovir was decreased (0.4%), which indirectly reflects tenofovir’s positive effect on the resistance to SARS-CoV-2 [18]. These findings are in agreement with the findings of our study. In addition, similar clinical findings in other viral diseases in patients with HIV have been reported. The study revealed a reduced SARS-CoV-2 diagnosis rate and improved COVID-19 prognosis in in HIV patients taking combination drugs including tenofovir and emtricitabine (TDF/FTC), among other protocols [19]. The author of the study suggested that tenofovir tended to produce the best overall COVID-19 outcomes. Experiments that have used molecular docking and extension reactions with RNA-dependent RNA polymerase (RNAdRNAp) also support the assertion that nucleos(t)ide reverse transcriptase inhibitors (NRTIs), such as tenofovir, abacavir, and lamivudine may be effective against SARS-CoV-2 by inhibiting RNAdRNAp [20]. Interestingly, TDF/FTC reduces SARS-CoV-2 titers in nasal washes from ferret infection models [21]. Tenofovir, recommended as a first-line treatment of CHB, has been described as having various immunomodulatory effects in human cell lines. COVID-19 infection has been reported to increase IL-6, interferon-γ, IL-10, and monocyte chemoattractant protein-1 levels [22]. Notably, tenofovir has been shown to diminish production of these inflammatory cytokines in monocytes and peripheral blood mononuclear cells [23]. However, we failed to demonstrate that antiviral treatment was associated with good prognosis in patients with SARS-CoV-2 and HBV coinfection. A possible reason may be the relatively small sample size with only 50 patients (0.6%) treated with antiviral agents in our study. Hence, these results should be interpreted with caution and further conclusive research is needed.

Findings of this report must be received with caution due to several limitations of this study. First, most patients were included based on medication prescriptions. In reality, medication history present in the electronic health record may not reflect actual drug exposure. The duration of antiviral treatment was ascertained. However, most antiviral drugs for CHB are taken for extended periods after initial administration, so defining treatment as two or more prescriptions is not a problem, and patients cannot take antiviral drugs without prescription in Korea. Another study limitation was that we used a large volume of data from a nationwide cohort sample; however, we could not guarantee that most of the population included had been exposed to SARS-CoV-2. Thus, our result may include selection bias. However, this study was designed with sufficient propensity score matching to overcome bias. Also, this nationwide cohort lacks history detailed laboratory information regarding the disease status of chronic hepatitis B (e.g., HBV DNA level, hepatic fibrosis stages). Given that the stages in patients with CHB are ambiguous (immune tolerance or low viral replication), these findings need to be confirmed in further studies. Finally, we could not adjust for several potential confounders including individual hygiene (i.e. hand washing), social distancing, and mask wearing, which may have impacted our findings.

Despite its limitations, to the best of our knowledge, this is the first large-scale study to investigate the association between COVID-19 risk and both underlying CHB and the use of antiviral therapeutics. Our results suggest that risk of SARS-CoV-2 infection is lower in patients with CHB than in the general population and that antiviral treatment including tenofovir reduced the risk of COVID-19. These findings suggest the host’s immune status is affected by CHB, to some extent, which may affect outcomes of SARS-CoV-2 infection and warrant further investigation using randomized controlled studies that assess effects of antiviral agents including tenofovir for the treatment and prevention of COVID-19.

## Supporting information

S1 File (DOCX)

## Abbreviations

aOR: adjusted odds ratio
CHB: chronic hepatitis B
COVID 19: coronavirus disease 2019
ICD-10: International Classification of Diseases, 10th revision
NHIS: National Health Insurance Service
OR: odds ratio
PS: propensity score
RNAdRNAp: RNA-dependent RNA polymerase
SARS-CoV-2: severe acute respiratory syndrome coronavirus 2
TDF/FTC: tenofovir and emtricitabine
