# Supplementary material for: Association between chronic hepatitis B infection and COVID-19 outcomes: A Korean nationwide cohort study
Source: PLoS One. 2021 Oct 5;16(10):e0258229. doi: 10.1371/journal.pone.0258229 (PMC8491877; doi:10.1371/journal.pone.0258229)
Supplement: S1 File — (DOCX) [file pone.0258229.s001.docx]

**Supplementary Documents**

**Association between chronic hepatitis B infection and COVID-19 outcomes: a Korean nationwide cohort study**

Seong Hee Kang^1,2¶^, Dong-Hyuk Cho^3¶^, Jimi Choi^4^, Soon Koo Baik^1,2^, Jun Gyo Gwon^5*^ and Moon Young Kim^1,2*^

**Contents**

| S1 Table. Association between history of CHB and risk of COVID-19^¶^ | p. 2 |
| --- | --- |
| S2 Table. Association between clinical outcomes of SARS-CoV-2 infection and CHB-associated cirrhosis | p. 4 |

**Supplementary Tables**

**S1 Table. Association between history of CHB and risk of COVID-19^¶^**

|  | **Total** | **N** | **(%)** | **Unadjusted OR** | **95% CI** | **P value** | **Adjusted OR** | **95% CI** | **P value** |
| --- | --- | --- | --- | --- | --- | --- | --- | --- | --- |
| **History of CHB** | |  |  |  |  |  |  |  |  |
| For COVID-19* |  |  |  | 0.57 | 0.50-0.66 | <.001 | 0.58 | 0.51-0.66 | <.001 |
| *Matched controls* | 45,571 | 2,482 | (5.4) |  |  |  |  |  |  |
| *Cases* | 7,690 | 267 | (3.5) |  |  |  |  |  |  |
| For death** |  |  |  | 1.52 | 0.84-2.75 | 0.168 | 1.16 | 0.59-2.29 | 0.662 |
| *Survival* | 7,455 | 255 | (3.4) |  |  |  |  |  |  |
| *Death* | 235 | 12 | (5.1) |  |  |  |  |  |  |
| For severity** |  |  |  | 1.66 | 1.10-2.52 | 0.016 | 1.25 | 0.80-1.96 | 0.336 |
| *Mild* | 7,212 | 241 | (3.3) |  |  |  |  |  |  |
| *Severe* | 478 | 26 | (5.4) |  |  |  |  |  |  |

**^¶^** Multiple conditional logistic regression analysis was performed without adjustment for cirrhosis, except for patients with other causes of cirrhosis

*By multiple conditional logistic regression model including hypertesion, DM and dyslipidemia as independent variables

**By multiple logistic regression model including age, gender, hypertesion, DM and dyslipidemia as independent variables

Abbreviations: CHB, chronic hepatitis B; CI, confidence interval; OR, odds ratio

**S2 Table. Association between clinical outcomes of SARS-CoV-2 infection and CHB-associated cirrhosis**

|  | **Total** | **N** | **(%)** | **Unadjusted OR** | **95% CI** | **P value** | **Adjusted OR** | **95% CI** | **P value** |
| --- | --- | --- | --- | --- | --- | --- | --- | --- | --- |
| **History of liver cirrhosis** | |  |  |  |  |  |  |  |  |
| For COVID-19* |  |  |  | 0.59 | 0.29-1.19 | 0.138 | 0.56 | 0.26-1.22 | 0.145 |
| *Matched controls* | 2,482 | 508 | (20.5) |  |  |  |  |  |  |
| *Cases* | 267 | 37 | (13.9) |  |  |  |  |  |  |
| For death** |  |  |  | 2.17 | 0.56-8.41 | 0.264 | 1.01 | 0.19-5.39 | 0.999 |
| *Survival* | 255 | 34 | (13.3) |  |  |  |  |  |  |
| *Death* | 12 | 3 | (25.0) |  |  |  |  |  |  |
| For severity** |  |  |  | 2.59 | 1.01-6.68 | 0.049 | 1.47 | 0.50-4.38 | 0.487 |
| *Mild* | 241 | 30 | (12.5) |  |  |  |  |  |  |
| *Severe* | 26 | 7 | (26.9) |  |  |  |  |  |  |

*By multiple conditional logistic regression model including hypertesion, DM and dyslipidemia as independent variables

**By multiple logistic regression model including age, gender, hypertesion, DM and dyslipidemia as independent variables

Abbreviations: CI, confidence interval; OR, odds ratio
